# Microsurgical resection of intracranial meningiomas in patients aged 80 years old or more: A systematic review and meta-analysis

**DOI:** 10.1016/j.bas.2025.104201

**Published:** 2025-03-03

**Authors:** Simon Diaz, Marc Levivier, Nicolas Reyns, Constantin Tuleasca

**Affiliations:** a Lausanne University Hospital (CHUV), Neurosurgery Service and Gamma Knife Center, Lausanne, Switzerland; b University of Lausanne (UNIL), Faculty of Biology and Medicine (FBM), Switzerland; c Neurosurgery and Neurooncology Service, Centre Hospitalier Regional Universitaire de Lille, Roger Salengro Hospital, Lille, France; d Ecole Polytechnique Fédérale de Lausanne (EPFL, LTS-5), Lausanne, Switzerland

**Keywords:** Intracranial meningioma, Microsurgical resection, Mortality, Morbidity, Elderly

## Abstract

**Introduction:**

The incidence of intracranial meningiomas rises with advancing age, raising the question of whether similar surgical outcomes in morbidity and mortality can be expected compared to younger population. We conducted a systematic review and meta-analysis of research examining microsurgical outcomes in those aged 80 years or older.

**Methods:**

Using Preferred Reporting Items for Systematic Reviews and Meta-Analyses guidelines, we reviewed articles published between January 1995 and January 2024, referenced in PubMed or Embase. Inclusion criteria were peer-reviewed clinical studies of series reporting microsurgical resection of intracranial meningiomas in patients aged 80 years or more, written in English. Primary outcome was EOR (extend of resection), classified as tumor total resection versus partial resection. Secondary outcomes were morbidity and mortality.

**Results:**

Ten studies reported 690 patients. Total tumor resection was achieved in 88% of cases (range 85–91; I^2^ = 56; p heterogeneity = 0.02; p < 0.01). Tumor partial resection was achieved in 12% (range 9–15; I^2 = 56; p heterogeneity = 0.02 and p < 0.001). Postsurgical intracerebral hemorrhage was encountered in 1% of cases (range 0–2; I^2 = 69; p heterogeneity = < 0.01). Surgical mortality was encountered in 5% of cases (range 3–7; I^2 = 61; p heterogeneity = < 0.01).

**Conclusion:**

Current data suggests that achieving high total resection rates, up to 88%, is feasible. The surgical mortality rate was 5%. A key unresolved neurosurgical dilemma is whether to operate on patients over 80 years old. Future studies are essential to assess all relevant risk factors comprehensively.

## Highlights

• This study shows a total tumor resection of 88%.
• The mortality rate was abnormally high, 5 %.
• We suggested a subtotal resection followed by radiosurgery on the residual tumors to preserve neurological function.
• Future studies are essential to assess all relevant risk factors comprehensively.

## 1 Introduction

Primary intracranial neoplasms occur more frequently with age (Dobran et al., 2018; Mastronardi et al., 1995; Schwartz et al., 2020). Intracranial meningiomas represent 15–20% of the adult's intracranial tumors, while the incidence increases with age (Riffaud et al., 2007; Konglund et al., 2013). The incidence is less than four per 100.000 in those younger than 50 years and seven to 10 per 100.000 in those older than 50 years (D'Andrea et al., 2005).

With the increasing life expectancy of humans and the general availability of neuroimaging in the last 15 years, the observation of symptomatic and asymptomatic intracranial meningiomas in patients aged 80 years or more has continuously increased. Such is confronting the neurosurgeon to decide if very elderly patients (>80 years old) should benefice of surgical removal (Dobran et al., 2018; Riffaud et al., 2007; Sacko et al., 2007) or should be irradiated (Ruess et al., 2020) or observed (Niiro et al., 2000).

Operating on patients over 80 years old with meningiomas can be justified when the tumor poses a significant risk of neurological decline or reduced quality of life, as timely intervention can prevent complications like disability or dependency. Additionally, surgery in selected cases can reduce long-term healthcare and societal costs by minimizing the need for prolonged medical care or assistance.

Some prognostic scales have been descripted to determine which patients would better benefit the surgery and could help to take the decision (Konglund et al., 2013; Sacko et al., 2007; Caroli et al., 2005). Furthermore, the improving of surgical technique has led to increase the volume of surgeries performed in the elderly. No clear data shows if the mortality of more than 80 years old with an intracranial meningioma is higher compared to a younger population consequently the benefice of the surgical treatment remain unclear. Moreover, there are large differences in the reported perioperative mortality (1.8–45%) (Mastronardi et al., 1995; Awad et al., 1989; Cornu et al., 1990; McGrail and Ojemann, 1994).

In the present systematic review and meta-analysis, we aim at evaluating the morbidity and mortality of patients aged more than 80 years (very elderly patients), operated for intracranial meningiomas. Such would potentially help in decision-making of the rationality of surgery for such benign tumors in the context of the continuous aging population.

## 2 Methods

### 2.1 Article selection and data extraction

A systematic review and meta-analysis was performed on 6 databases by two independent reviewers (SD, CT): PubMed, Scopus, Ovid, Embase, Google Scholar, and clinicaltrial.gov between January 1995 and January 2024. We use a combination of different “mesh-words” for specific search (mesh) terms: “intracranial meningioma”, “elderly”, “microsurgery”, “mortality”, “survival”, “80 years old”, “aging”, “resection”, “morbidity”.

Inclusion criteria were studies evaluating the outcome of microsurgical resection of intracranial meningiomas in more than 80 years old patients; published either in English or French. Exclusion criteria were letters, case reports, studies without full text available, book chapters, and duplicate studies from the same group (s). Moreover, peer-reviewed articles evaluating other age groups (cut-off of 60 or 70's) were also excluded.

We began by screening the title and abstract and then continued with the full text to evaluate the potentially eligible studies, which meet the inclusion and exclusion criteria.

Two independent reviewers (SD, CT) assessed the data by applying the inclusion and exclusion criteria. There were no disagreements.

This study was performed in agreement with the published Preferred Reporting Items for Systematic Review and Meta-Analyses guidelines (Moher et al., 2009).

**Fig. 1:**
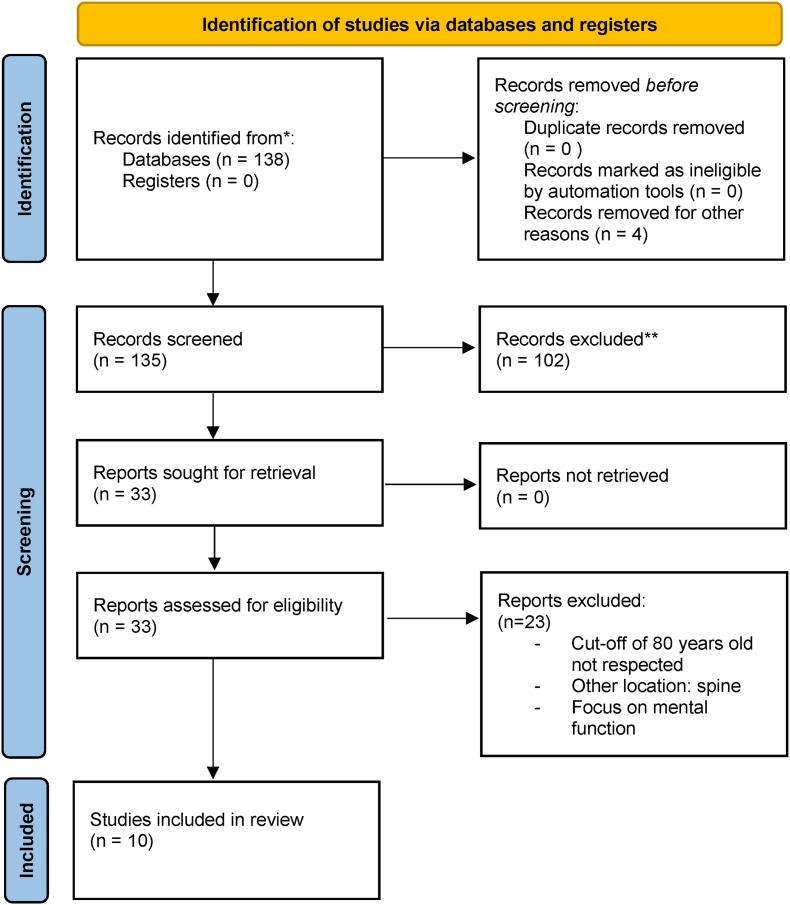
PRISMA flowchart. PRISMA 2020 flow diagram for new systematic reviews which included searches of databases and register only. ∗Consider, if feasible to do so, reporting the number of records identified from each database or register searched (rather than the total number across all databases/registers). ∗∗If automation tool were used, indicate how many records were excluded by a human and how many were excluded by automation tools.

Data extraction was done as per individual studies (Fig. 1, Table 1).

**Table 1:** Basic demographic data.

	Numbers of cases	Age (range)	Sex (M:F)	Location	KPS	ASA (class)	Indications for surgery
(Mastronardi et al., 1995)	17	80–86	4:13	10 convexity	≥70 = 10	I: 2	–
				4 parasagittal	60 = 3	II: 11	
				3 cranial base	50 = 4	III: 4	
(D'Andrea et al., 2005)	37	80–86	8:29	23 convexity	>70 = 23	I: 11	Intracranial hypertension: 27/37 (73%)
				7 parasagittal	<70 = 10	II: 19	Gait difficulty: 17/37 (46%)
				1 posterior fossa	<60 = 4	III: 7	Seizure: 16/37 (43%)
				3 sphenoidal ridge			Sensory loss: 10/37 (27%)
				1 tuberculum			Paresis: 11/37 (30%)
				2 orbitocranial			Language problems: 6/37 (16%)
							Visual loss: 5/37 (13%)
							Cerebellar symptoms: 1/37 (3%)
(Riffaud et al., 2007)	11	81–87	5:6	9 convexity	>80 = 3	I: 0	Language problem: 6/11 (54%)
				1 cranial base	>40 = 8	II: 8	Paresis: 3/11 (27%)
				1 ventricular		III: 3	Gait difficulty: 1/11 (9%)
							Visual loss: 1/11 (9%)
(Sacko et al., 2007)	74	80–90	27:47	35 convexity	–	–	Seizure: 32/74 (43%)
				6 parasagittal			Intracranial hypertension: 16/74 (22%)
				7 falx			Language problems: 12/74 (16%)
				4 olfactory groove			Cerebellar symptoms: 12/74 (16%)
				4 sphenoidal ridge			Paresis: 48/74 (65%)
				11 infratentorial			
				3 multiple meningioma			
(Konglund et al., 2013)	51	80–90	24:27	–	>80 = 21	I/II: 17	–
					60-70 = 21	III: 30	
					<50 = 9	IV: 4	
(Steinberger et al., 2018)	93	–	–	–	–	–	–
(Dobran et al., 2018)	25	80–87	8:17	16 convexity	21 > 70	II: 15	Neurological deficits without further details: 21/25 (84%)
				5 parasagittal/falx	4 < 70	III: 10	Seizure: 4/25 (16%)
				4 cranial base			
(Rautalin et al., 2021a)	83	80–96	26:57	31 convexity	Median 80	I: 0	Paresis: 18/83 (22%)
				9 falx		II: 5	Gait difficulty: 10/83 (12%)
				33 cranial base		III: 44	Visual loss: 9/83 (11%)
				7 posterior fossa		IV: 34	Seizure: 5/83 (6%)
				3 others			Language problems: 2/83 (2%)
(Joubert et al., 2021)	37	80–93	17:20	16 convexity	>70 = 15	I: 8	Paresis: 17/37 (46%)
				9 parasagittal	60-70 = 17	II: 16	Seizure: 15/37 (41%)
				6 middle skull base	<60 = 5	III: 13	Language problems: 7/37 (19%)
				4 anterior skull base			Intracranial hypertension: 4/37 (11%)
				2 posterior fossa			Sensory loss: 4/37 (11%)
							Visual loss: 1/37 (3%)
(Schwartz et al., 2024)	262	80–96	150:112	93 convexity	>80 = 117	–	Neurological deficit/mass effect: 242/262 (92%)
				61 sphenoid	50 -70 = 113		Other/unspecified: 20/262 (8%)
				37 falx	<40 = 32		
				37 anterior skull base			
				27 middle skull base			

### 2.2 Primary and secondary outcomes

Primary outcome was the extent of resection (EOR). Particular attention was paid to tumor total resection and partial resection. We conventionally defined total resection as complete or Simpson I or II microsurgical resection, while partial resection as anything defined as subtotal, to be able to report uniformly and with enough statistical power such results (please see Table 2).

**Table 2:** Postoperative results.

	Total:subtotal resection	WHO grade	Mortality (1–3 months)	Intracranial hemorrhage	Wound problem and deep infection	Other distant infections	Seizure	Pulmonary embolism	CSF leak
(Mastronardi et al., 1995)	13:4	–	4 (29.4%)	–	1/17 (5.9%)	–	–	–	1/17 (6%)
(D'Andrea et al., 2005)	30:7	–	5 (13.5%)	–	–	–	–	–	1/37 (3%)
(Riffaud et al., 2007)	11:0	–	0	–	–	–	–	–	–
(Sacko et al., 2007)	61:13	I: 47	1 (1.4%)	–	–	–	–	–	–
		II: 22							
		III: 5							
(Konglund et al., 2013)	–	I: 45	2 (3.9%)	7/51	2/51 (4%)	14/51 (27%)	–	–	–
		II: 5							
		III: 1							
(Steinberger et al., 2018)	–	–	8 (8.6%)	–	1/93 (1%)	13/93 (14%)	–	7/93 (8%)	–
(Dobran et al., 2018)	18:7	I: 17	2 (8%)	–	1/25 (4%)	–	–	–	2/25 (8%)
		II: 8							
(Rautalin et al., 2021a)	77:6	I: 52	6 (7%)	8/83	–	5/83 (6%)	5/83 (6%)	–	–
		II: 19							
(Joubert et al., 2021)	34:3	–	1 (2.7%)	4/37	–	–	1/37 (3%)	–	–
(Schwartz et al., 2024)	225:37	I:180	24 (9%)	11/262	6/262	14/262	12/262	–	–
		II: 76							
		III: 6							

The research question in PICO format was: in very elderly patients, aged 80 years old or more (P), what is the extent of resection (O) when treated with microsurgical resection (I), without a comparison group (C)?

Secondary outcome was morbidity and mortality due to the microsurgical resection. We defined major morbidity as one related to symptomatic intracranial hemorrhage. Other complications were scarcely reported among the studies and included diffuse cerebral edema, cardio-respiratory failure, pneumonia, infection, seizure, CSF leak and pulmonary embolism. For more details, please see Table 2. Not all studies reported all outcomes. In this respect, each total number of cases might be different as per separate outcome.

### 2.3 Statistical analysis

For our meta-analysis, only the studies reporting the individual data were analyzed. Because of high variations in study characteristics, a statistical analysis using a binary random-effects model (DerSimonian–Laird method) was performed using OpenMeta[analyst] software (Agency for Healthcare Research and Quality). Weighted summary rates were determined using meta-analytical models. Heterogeneity was tested for each meta-analysis; pooled estimates were obtained for all outcomes. Results of series concerning EOR and morbidity were compared using a meta-regression with a random effect. *P* values < 0.05 were considered statistically significant.

## 3 Results

### 3.1 Indications for microsurgical resection

The indications for microsurgical resection were: intracranial hypertension, seizure, visual loss, gait difficulties, language problems, cerebellar symptoms, sensory loss, paresis (for more detailed information please see Table 1).

## 4 Study selection and patient population

We finally report 10 series reporting a total number of 690 patients (Dobran et al., 2018; Mastronardi et al., 1995; Schwartz et al., 2020; Riffaud et al., 2007; D'Andrea et al., 2005; Sacko et al., 2007; Joubert et al., 2021; Rautalin et al., 2021a; Rautalin et al., 2021b; Steinberger et al., 2018; Schwartz et al., 2024).

## 5 Tumor control

### 5.1 Tumor total resection

**Fig. 2:**
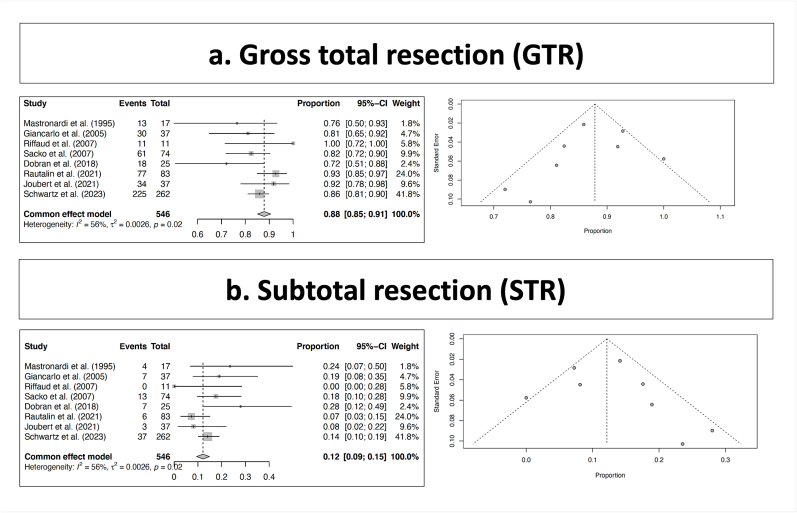
Tumor control.

Tumor total resection was encountered in 469 out of the 546 reported patients, which corresponded to a rate of 88% (range 85–91; I^2 = 56; p heterogeneity = 0.02 and p < 0.001); Fig. 2, a; Table 2).

### 5.2 Tumor partial resection

Tumor partial resection was encountered in 77 out of the 546 reported patients, which corresponded to a rate of 12% (range 9–15; I^2 = 56; p heterogeneity = 0.02 and p < 0.001); Fig. 2, b; Table 2).

## 6 Major morbidity and mortality

### 6.1 Intracerebral hemorrhage

**Fig. 3:**
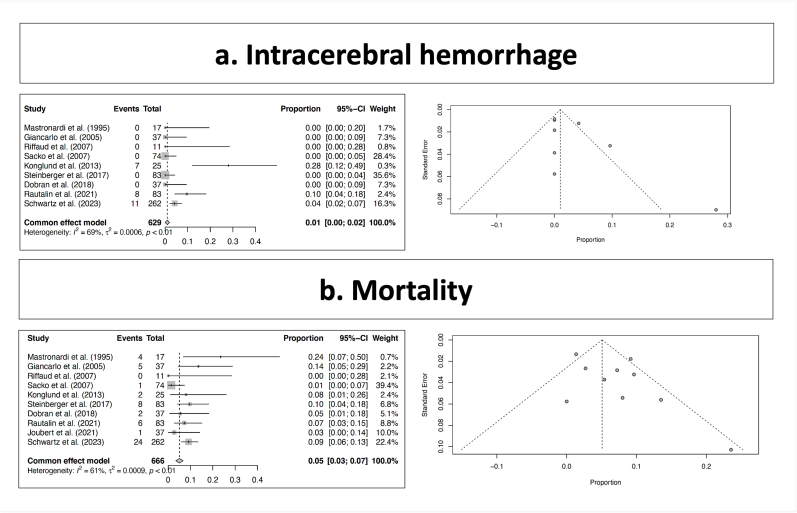
Major morbidity and mortality.

Postsurgical intracerebral hemorrhage was encountered in 26 out of the 629 reported patients, which corresponded to a rate of 1% (range 0–2; I^2 = 69; p heterogeneity = < 0.01; Fig. 3, a; Table 2).

### 6.2 Mortality

Surgical mortality was encountered in 53 out of the 666 reported patients, which corresponded to a rate of 5% (range 3–7; I^2 = 61; p heterogeneity = < 0.01; Fig. 3, b; Table 2).

## 7 Discussion

Our current systematic review and meta-analysis unveiled a noteworthy rate of complete tumor resection, reaching as high as 88%. The incidence of postsurgical intracerebral hemorrhage was relatively low, affecting only 1% of patients. The mortality rate was recorded at 5%.

Making operative decisions for a patient older than 80 years can be challenging. Over the past decades, various scales have been developed to assess whether surgery would be beneficial for elderly individuals, as, for example, the SKALE score by Sacko et al. (2007), which includes sex, tumor location, tumor removal, tumor size, KPS score, ASA class and peritumoral edema. It was further recommended that near-total removal of intracranial meningiomas should be attempted in very elderly patients if the SKALE score is 8 or greater (Konglund et al., 2013; Sacko et al., 2007). Caroli et al. (2005) proposed a clinical radiological grading scale using six factors, including lesion size, neurological condition, KPS, location, edema and concomitant disease so as to determine the outcome and concluded that female sex and a higher score were associated with a higher probability of survival (Caroli et al., 2005). Steinberger et al. (2018) suggested that age over 80 years is an independent risk factor for death within 30 days of surgery following craniotomy for resection of meningiomas (Steinberger et al., 2018). Despite numerous studies, a consensus regarding the surgical management of intracranial meningiomas in very elderly patients has not been reached. However, nowadays, there is a growing consensus that complex neurosurgical procedures can be successfully performed in very elderly patients with acceptable outcomes (Riffaud et al., 2007). Particularly and after microsurgical resection, quality of life was improved (Mastronardi et al., 1995; Riffaud et al., 2007; D'Andrea et al., 2005; Caroli et al., 2005; Tucha et al., 2001). Pre-operative KPS and ASA score were linked to an overall worst post-operative outcome in most studies (Dobran et al., 2018; Mastronardi et al., 1995; Riffaud et al., 2007; Konglund et al., 2013; D'Andrea et al., 2005; Sacko et al., 2007). Regarding location, the extent of resection and severity of the edema, the literature is contradictory with influence on the mortality rate (Mastronardi et al., 1995; Schwartz et al., 2020; Riffaud et al., 2007; D'Andrea et al., 2005; Sacko et al., 2007; Rautalin et al., 2021a).

An open question is whether total resection should be aim in this population of patient, particularly in the context of the age, the remaining time for a potential tumor growth and the possibility to apply stereotactic radiosurgery on the tumor residue, if necessary. In our opinion, we do believe that subtotal resection should be the aim, with preservation of neurological function.

Concerning the microsurgical duration of the procedure, there is no consensus among studies regarding its relationships with the mortality. Dobran et al. (2018) found that the surgical duration seems to be the most important prognostic factor for the outcome of elderly patients. Based on our studies, surgical removal of IMs with larger peritumoral edema associates with more favorable outcome whereas surgical treatment of giant (diameter ≥5 cm) intracranial meningiomas entails a high complication rate (Rautalin et al., 2021a). Complication rates were very variable from one study to the other.

The results of this study highlight a discrepancy between the hemorrhage rate of 1% and mortality of 5%. This is also a limitation due to the fact that not all studies specifically reported and detailed the individual preoperative comorbidities and further postoperative complications. In this respect, to arrive at a mean of 5% mortality rate, other perioperative and postoperative events appeared, other than hematoma, to explain such numbers. A higher risk of postoperative mortality was linked to the worst preoperative general conditions, usually when the preoperative KPS rating was 60 or lower (D'Andrea et al., 2005), while it was not influenced by size and location of the meningioma, the extent of surgical removal, and the severity of peritumoral edema. Sacko et al. suggested that critical location and severe edema were associated with high mortality in the first year after surgery (Sacko et al., 2007). Konglund et al. (2013) suggested that the SKALE score reflects the mortality at both 1 month and 1 year following primary surgery for intracranial meningiomas in our very old patients and might further represent a helpful adjunct to their preoperative assessment. Overall, mortality rates tend to increase with age across studies. However, advancements in neurosurgical techniques, along with improved postoperative nursing care, have contributed to a simultaneous decrease in mortality. Rautalin and al(Rautalin et al., 2021c). previously conducted a systematic review on surgical outcomes for intracranial meningioma in patients aged 80 years and older, incorporating studies up to 2018. Their findings indicated that surgery is relatively safe for this patient group, with mortality rates ranging from 0 to 23.5% at 1 month to 9.4–27.3% at 1-year post-operation. However, our study incorporates five more studies. Moreover, we enhance the existing research with a meta-analysis that provides data consistent with their findings. We also included the recent study by Schwartz et al. (2024), with a total number of 262 patients and a 90-day and 1-year mortality rate of 9.0% and 13.2%, which is much higher than reported here.

Our study has several inherent limitations. The first is related to the retrospective nature of the included studies. The second reflects the different anatomical locations, with some (i.e. convexity) being more easily amenable to complete microsurgical resection. Long-term control was not provided; furthermore, the follow-up length was extremely variable. With regards to some studies, one can see that some of the 95% CIs are wide; as an example, Mastronardi paper for GTR an STR. Such can be explained by smaller sample size (17 patients for Matronardi, 25 for Dobran concerning GTR/STR), as studies with limited number of patients are more prone to random variation that can impacts significantly the rates outcome. Another limitation was the heterogeneity in health status and physiological resilience among individuals aged 80 years or older.

## 8 Conclusion

The open question is whether to operate on a patient older than 80 years is an important and difficult neurosurgical quest. Our study suggests a high rate of total tumor resection, as high as 88%. Postsurgical intracerebral hemorrhage was encountered in a low percentage of 1% of patients. Mortality rate was 5% which is abnormally high. Future studies are needed to comprehensively assess all relevant risk factors. Anatomic location and tumors adhesion to the brain parenchyma remains critical factors for pre-operative and intra-operative assessment. Depending on the previous, we would advocate for subtotal resection followed by stereotactic radiosurgery. Further prospective and randomized control trial are needed to confirm such finding.

## Consent to participate

Not applicable.

## Availability of data and material

Not applicable.

## Code availability

Not applicable.

## Authors' contributions

All authors contributed to the study conception and design. The first draft of the manuscript was written by Simon Diaz and Constantin Tuleasca and all authors commented on previous versions of the manuscript. All authors read and approved the final manuscript.

## Ethical approval

Not applicable.

## Consent for publication

Not applicable.

## Funding

No funding was received for this research.

## Declaration of competing interests

All authors certify that they have no affiliations, with or involvement in any organization or entity with any financial interest (such as honoraria; educational grants; participation in speakers' bureaus; membership, employment, consultancies, stock ownership, or other equity interest; and expert testimony or patent-licensing arrangements), or non-financial interest (such as personal or professional relationships, affiliations, knowledge or beliefs) in the subject matter or materials discussed in this manuscript.
